# Sodium thiosulfate (hydrogen sulfide donor) ameliorates the pancreatic and liver damage induced by cyclophosphamide and/or ionizing gamma radiation in male albino rats

**DOI:** 10.1186/s40360-025-01011-0

**Published:** 2025-10-29

**Authors:** Ashraf Kassem, Eman F. S. Taha, Azza Hassan, Marwa A. Ibrahim, Ahmed H. Osman

**Affiliations:** 1 Department of Pathology, Faculty of Veterinary Medicine, Cairo University, Al Giza, Egypt; 2 Health Radiation Research Department, National Center for Radiation Research and Technology (NCRRT), Egyptian Atomic Energy Authority, Cairo, Egypt; 3 Department of Biochemistry and Molecular Biology, Faculty of Veterinary Medicine, Cairo University, Al Giza, Egypt

**Keywords:** Sodium thiosulfate, Ionizing gamma radiation, Cyclophosphamide, Histopathology, Immunohistochemistry

## Abstract

**Background:**

Radiotherapy and cyclophosphamide (CYP) treatment can adversely affect various organs, including the liver and pancreas. In addition to hepatic toxicity, CYP and/or ionizing gamma radiation (R) may impact the exocrine and endocrine functions of the pancreas. This study aims to determine whether sodium thiosulfate (STS) can protect male rats against CYP and/or R-induced damage to the pancreas and liver.

**Materials and methods:**

Sixty-four rats were divided into eight groups, with STS administered for 14 days prior to CYP and/or R treatment. Enzymatic activities of alanine aminotransferase (ALT) and aspartate aminotransferase (AST), as well as insulin and glucose levels, were assessed. Amylase and lipase concentrations were measured using enzyme-linked immunosorbent assay (ELISA), and malondialdehyde (MDA) levels were determined to evaluate lipid peroxidation. Glutathione content (GSH), glutathione-S-transferase (GST) activity, and reactive oxygen species (ROS) were quantified. Gene expression of extracellular signal-regulated kinase (Erk-1) and c-Jun N-terminal kinase (JNK) was analyzed using real-time quantitative polymerase chain reaction (qPCR). Histopathological examination and immunohistochemical staining with anti-nuclear factor erythroid 2-related factor 2 (anti-Nrf2) antibodies were performed.

**Results:**

STS treatment increased GSH, GST, and insulin levels while reducing ROS, MDA, glucose, amylase, and lipase levels. Furthermore, the STS treatment significantly downregulated mitogen-activated protein kinases (MAPKs) such as ERK and JNK Additionally, STS administration increased Nrf2 levels in both pancreatic and hepatic tissues while minimizing pathological changes. These findings suggest that STS may hold promise as a protective agent against CYP and/or R-induced liver and pancreatic damage.

**Conclusion:**

The ability of STS to enhance antioxidant defenses, reduce oxidative stress, modulate signaling pathways, and preserve tissue integrity suggests its therapeutic potential in mitigating the detrimental effects of these damaging conditions by inhibiting the MAPK, ERK, and JNK signaling pathways.

## Introduction

Pancreatic and liver injuries are significant concerns associated with cancer treatments, particularly chemotherapy and radiotherapy. CYP, a widely used chemotherapeutic agent, and R are known to cause detrimental effects on pancreatic and liver tissues. These adverse effects can lead to compromised organ function, impaired metabolism, and reduced overall health outcomes in cancer patients [1]. CYP, an alkylating agent, induces oxidative stress, inflammation, and DNA damage across various tissues. Similarly, exposure to R generates ROS, triggers inflammatory responses, and disrupts cellular homeostasis. The pancreas and liver, being highly metabolic organs, are particularly vulnerable to the damaging effects of CYP and radiation, which can disrupt their intricate cellular architecture and impair crucial physiological functions [2].

Cancer is a complex disease that presents a wide spectrum of symptoms in both humans and animals. Treatment options include surgery, biological therapy, immunotherapy, and chemotherapy. Among these, chemotherapy remains one of the most widely used modalities, particularly effective for various cancers such as lymphoma, leukemia, and solid tumors including lung, breast, and ovarian cancers [3].

CYP is a commonly utilized chemotherapeutic agent that is metabolized by cytochrome P450 enzymes into phosphoramide mustard and acrolein. Acrolein plays a significant role in contributing to oxidative stress through the generation of ROS [4]. The toxicity associated with CYP is characterized by several factors: depletion of GSH, lipid peroxidation, alterations in DNA profiles, a pro-inflammatory response, and induction of apoptosis [5]. These mechanisms highlight the intricate balance between the therapeutic effects of chemotherapy and its potential toxicities.

Radiotherapy can be beneficial for patients with hepatocellular carcinoma (HCC) and other liver disorders. However, radiation exposure—whether accidental, occupational, or therapeutic—can lead to tissue reactions that result in both direct and indirect alterations [6]. A common and serious side effect of abdominal irradiation is acute pancreatic damage, which disrupts DNA, proteins, and lipids in mammalian cells, triggering apoptosis and stress-related responses [7]. Moreover, radiation exposure during gastrointestinal cancer treatment can harm healthy liver tissue, with excessive ROS production emerging as a key mechanism promoting pancreatic deterioration and hepatic fibrosis following radiation [8].

Both CYP and R can activate various MAPKs, with particular emphasis on extracellular ERK and JNK [9]. These pathways can lead to significant alterations in cellular transcription and function. STS, H_₂_S donor, has been shown to protect pancreatic β-cells by decreasing ROS production and increasing GSH levels, thereby mitigating oxidative stress [10].

Recent studies have focused on potential therapeutic interventions aimed at alleviating the toxic effects of chemotherapy and radiation. STS has gained attention for its antioxidant, anti-inflammatory, and cytoprotective properties [11]. Preclinical studies suggest that STS can mitigate oxidative stress, reduce inflammation, and preserve cellular integrity in various tissues [2]. However, the specific effects of STS on pancreatic and liver tissues in the context of CYP and radiation-induced damage remain relatively unexplored [12].

Furthermore, STS has been shown to regulate the enzymatic and hormonal secretions of the pancreas, including amylase, lipase, and insulin [13]. Recent findings indicate that exogenous STS ameliorates hepatic tissue damage and reduces serum AST and ALT levels by inhibiting ROS and MDA production induced by CYP and R [14]. Additionally, STS suppresses the activation of MAPKs, which play critical roles in cell proliferation, survival, and apoptosis [15]. The pleiotropic effects of STS, encompassing antioxidant, anti-inflammatory, and anti-apoptotic properties, further underscore its therapeutic potential [16].

Understanding the mechanisms underlying the protective effects of STS may pave the way for the development of novel strategies to mitigate the adverse effects of chemotherapy and radiation therapy, ultimately enhancing patient outcomes and improving the overall quality of life for individuals undergoing cancer treatment.

## Materials and methods

### Reagents and chemicals

CYP injection (Endoxan-N 1 g) was purchased from a general pharmacy; STS (Chem Center 5580 La Jolla Blvd., San Diego, CA); and all other chemicals were of analytical grade and were purchased from Sigma Aldrich Chemical Co. (St. Louis, Missouri, USA).

### Radiation research facility

A cell-40 biological irradiator unit at the National Center for Radiation Research and Technology (NCRRT) in Cairo, Egypt, utilized gamma-cesium-137 radiation, supported by a radiation resource from Atomic Energy of Canada ltd. Ottawa, Ontario, Canada. This setup ensured a homogeneous dose distribution across the irradiation tray [17]. The rats were exposed to R from a distance of 1 m from the source, with the radiation delivered in two parts—half from above and half from below [18]. A radiophotoluminescence glass dosimeter (GD-302 M; Chiyoda Technol Co., Tokyo, Japan) was used to measure the dose [19]. Rats were not anesthetized during the radiation procedure due to the short exposure duration and non-invasive setup, which allowed for adequate restraint without compromising their safety or comfort. After being placed inside a specially designed acrylic container with adequate positioning and ventilation, aligning with protocols where anesthesia is unnecessary for brief, well-controlled procedures [20]. Rats were exposed to whole-body fractionated doses of 3 Gy/week up to a total cumulative dose of 9 Gy of R (3 Gy x 3 weeks) at a dose rate of 0.33 Gy/min at the time of the experiment as described in the previous studies with slight modifications [21–23].

### Experimental animals

Sixty-four male Wistar albino rats, weighing between 250 and 350 g, were procured from the Veterinary Medicine Cairo University breeding unit. Prior to commencing the experiment, these animals underwent a one-week acclimatization period to standard laboratory conditions, featuring a temperature range of 21–23 °C, a relative humidity level of 50 ± 5%, and a 12-hour light-dark cycle. These conditions were consistently maintained throughout the study, aligning with the guidelines on the Care and Use of Laboratory Animals [24]. All procedures were conducted in compliance with the pertinent guidelines and regulations, adhering to the ARRIVE guidelines for the care and utilization of experimental animals set forth by the Committee for the Purpose of Control and Supervision of Experiments on Animals (CPCSEA) and the protocol of the National Institutes of Health (NIH) [25]. The described methodologies were thoroughly reviewed and approved by the Veterinary Medicine Cairo University Institutional Animal Care and Use Committee (Vet. CU. IACUC; registration number: vet cu 2305-2022451).

### Experiment design

**Fig. 1:**
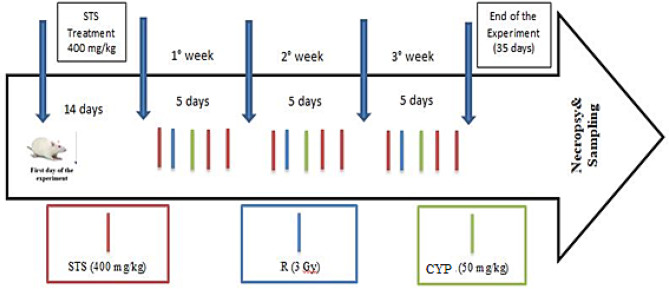
Experimental design

The rats were divided into eight groups (*n* = 8), as follows: **Group 1 (Control)**: Rats were treated with 0.5 mL of normal saline (i.p.) for 5 successive weeks. **Group 2 (STS)**: Based on previous studies [12, 26–28], rats were treated with STS (400 mg/kg/day i.p.) at a constant dose of 5 mL/kg body weight for 5 weeks. **Group 3 (CYP)**: Rats were treated with fractionated doses of CYP (50 mg/kg/week, i.p.) for three successive weeks, up to a total cumulative dose of 150 mg/kg. **Group 4 (R)**: Rats were exposed to R (3 Gy/week up to a cumulative dose of 9 Gy/experimental course) for three successive weeks. **Group 5 (CYP + R)**: Rats were treated with CYP as in group 3 and exposed to R as in group 4. The CYP was administered 24 h after R each week in the experiment. **Group 6 (STS + CYP)**: Rats were treated with STS as in Group 2, then three times per week for three successive weeks during CYP treatment. **Group 7 (STS + R)**: Rats were treated with STS as in Group 2, then three times per week for three successive weeks during R treatment. **Group 8 (STS + CYP + R)**: Rats were treated with STS as in Group 2, then three times per week during CYP and R treatments. Rats will be subjected to R 24 h before CYP administration each week of the experiment (Fig. 1).

### Anesthesia and euthanization of rats

Rats were anesthetized using an intraperitoneal injection of urethane at a dosage of 1.5 g/kg body weight [29], with the depth of anesthesia monitored by assessing the lack of response to noxious stimuli. The euthanasia of animals was carried out according to the American Veterinary Medical Association (AVMA) Guidelines for the Euthanasia of Animals (2020) [30]. Euthanasia was performed via cervical dislocation by trained personnel to ensure a humane and rapid procedure [31]. Confirmation of death was achieved through the absence of response to noxious stimuli (e.g., toe pinch), lack of respiratory activity, and absence of heartbeat, ensuring that euthanasia was conducted ethically and in accordance with established guidelines [32].

### Sample collection

Following standard laboratory procedures, blood samples were collected from rats under anesthesia via heart puncture and placed in serum vacuum tubes. The blood was centrifuged at 1000 g for 10 min after it had coagulated [33]. Following euthanasia, liver and pancreas samples were carefully collected and rinsed with ice-cold PBS to remove contaminants. To make a 10% (w/v) tissue homogenate for biochemical analysis, a portion of the liver and pancreas were homogenized in PBS buffer (0.1 M, pH 7.4) [34]. For gene expression studies, portions of both tissues were immediately snap-frozen in liquid nitrogen to preserve RNA integrity and stored at -80 °C. Prior to RNA extraction, these frozen tissues were pulverized into a fine powder under liquid nitrogen using a mortar and pestle and homogenized in TRIzol reagent [35]. The remaining pancreas and liver tissues were fixed in 10% neutral buffered formalin for histopathological examination and immunohistochemical analysis.

### Measurement of biochemical parameters in the serum

The activities of ALT and AST were determined according to King’s method [36]. In addition, insulin and glucose levels were evaluated by the enzymatic method described by Kurahashi and Inomata [37]. Rat serum pancreatic alpha-amylase and pancreatic lipase concentrations were measured using commercially available ELISA kits according to the manufacturers’ protocols. Specifically, the Rat Alpha Amylase ELISA Kit (Catalog Number: MBS3809242) and the Rat Lipase ELISA Kit (Catalog Number: MBS3808576) were both procured from MyBioSource, USA.

### Determination of oxidative stress

Pancreatic and liver levels of MDA, a marker of lipid peroxidation, were quantified using the method outlined by Uchiyama and Mihara [38]. The determination of GSH content was performed as described in Staal et al. [39]. Additionally, the activity of GST was measured following the protocol outlined in Habig et al. [40]. The quantification of ROS was conducted according to the procedure described by Vrablic et al. [41].

### Real-time quantitative polymerase chain reaction determination of Erk-1 and JNK gene expression

Using the Qiagen mini-RNeasy extraction kit according to the manufacturer’s recommendations, total RNA was extracted from the pancreas and liver samples. The concentration and purity were determined using spectrophotometry at 260 and 280 nm. Then, complementary DNA (cDNA) was created using a Revert Aid First Strand cDNA Synthesis Kit (Thermo Scientific, USA) following the manufacturer’s instructions after employing DNase I to remove DNA contamination (Fermentas, Lithuania). Using the Rattus Novice sequences found in GenBank, primer sets for detecting mRNA levels of specific genes were designed (Table 1). The primer sets were built using the Primer tool. We employed SYBR Green PCR Master Mix, and real-time PCR analysis was performed to evaluate the relative expression of the chosen genes (Thermo Scientific Cat number: 4309155) using the ABI Prism Step One Plus Real-Time PCR System from Applied Biosystems as instructed by the manufacturer [42]. The PCR reactions were run twice on each sample. The expression levels of Jnk and ERK-1 were normalized to the housekeeping gene beta-actin. Using the delta-delta Ct (DDCt) technique, gene expression data were calculated.

**Table 1:**
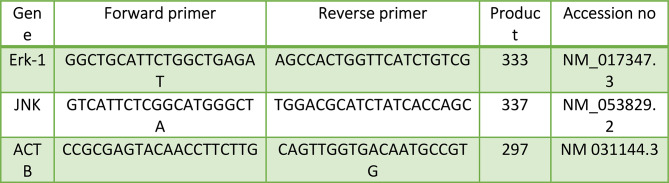
Primer sets for detecting ERK-1 and JNK mRNA levels

### Histopathological examination

Pancreatic and hepatic tissue specimens were collected from all groups at the end of the experiment and fixed in a 10% neutral buffered formalin solution for histopathology. Tissue specimens were processed as follows: dehydrated in an ascending ethanol concentration, cleared in xylene, embedded in paraffin wax, and sectioned at a 5 μm thickness. Prepared slide sections were stained with hematoxylin and eosin and examined by a light digital microscope (Olympus XC30, Tokyo, Japan) [43]. Apoptosis and/or single-cell necrosis in pancreatic acinar cells and islet cells were assessed semi-quantitatively in five random high microscopic power fields (40X) per group to assess pancreatic injury [44]. A three-scale grading system was used: Grade 0 = normal exocrine and endocrine pancreatic acinar cells; Grade 1 = sparse apoptosis; Grade 2 = few apoptosis and/or single cell necrosis; Grade 3 = numerous apoptosis and/or single cell necrosis. In addition, the endocrine pancreas diameter was measured in different treated groups to score regeneration capacity. On the other hand, the hepatocellular injury was semi-quantitatively assessed in ten random high-microscopic power fields (40X) using a grading system scaled from 0 to 3, as described [45]. The main pathological parameters used in this assessment were vacuolar degeneration of hepatocytes and apoptosis. Grade 0 = normal histological structure; 1 = mild hepatocellular swelling; 2 = moderate hepatocellular swelling with sparse apoptosis; and 3 = extensive swelling with abundant apoptosis.

### Immunohistochemical examination

Immunohistochemical staining of the pancreatic and liver tissues with anti-Nrf2, to investigate the protective effects of STS on oxidative stress, was performed according to the method of Saleh et al. [46]. After deparaffinization in xylene, the sections were rehydrated in alcohol and incubated in 3% H_2_O_2_. The sections were then incubated with rabbit monoclonal anti-Nrf2 (SAB4501984, China) as primary antibodies. Diaminobenzidine (DAB; Sigma, USA) was used to demonstrate the immune reactivity. The Nrf2 immunoreactivity was semi-quantitatively assessed in ten high microscopic power fields (40X) [47]. A total immunoreactivity score is used for the assessment of Nrf2 immunoreactivity. This score is based on the sum of two main criteria: the color intensity and the percentage of positively stained cells. Both the color intensity and the percentage of positively stained cells were graded on a scale of 0 to 3. In terms of color intensity, grade 0 indicates no staining, grade 1 indicates weak staining, grade 2 indicates moderate staining, and grade 3 indicates strong staining. On the other hand, in terms of the percentage (%) of positively stained cells, grade 0 indicates 0%, grade 1 indicates 30%, grade 2 indicates 30%–70%, and grade 3 indicates >70%.

### Statistical analyses

A statistical analysis of the obtained data was performed using Statistical Package for Social Sciences for Windows software (SPSS version 20.0, SPSS Inc., Chicago, USA). Raw data were statistically analyzed for the normal distribution using the Shapiro-Wilk test. The mean and standard error of descriptive statistical values were shown (*n* = 8 per group). For parametric tests, the one-way analysis of variance (ANOVA), post hoc, and Tukey tests were used to evaluate group differences, while Kruskal-Wallis, a pairwise comparison with post-hoc Dunn’s test, were used for non-parametric analyses of the histopathological and IHC staining scores. All data were expressed as mean (if distributed normally) or median (if not distributed normally) plus standard error (SE), interquartile range (IQR), or individual data points. P-value (p) < 0.05 was considered significant. The number of rats used per group was calculated using GPower 3.1.9.4 (Heinrich Heine University Düsseldorf, Dusseldorf, Germany) [48]. Taking into consideration our preliminary results, we have considered an effect size of 0.94. Determining, based on significance level (α) at 0.05 and testing power (1-β) at 0.8 for 8 groups, a total of 64 adult male Wistar rats was determined (8 rats/group).

## Results

### Effect of STS on serum hepatic biomarkers in CYP and/or R-treated groups

The study revealed significant alterations in liver enzyme levels, as reflected by changes in ALT and AST compared to the control group. In the STS group, ALT and AST levels showed a slight increase of approximately 2.3% and 4.3%, respectively, relative to the control group. These changes were not statistically significant compared to the control group (*p* > 0.05) but were significantly lower than those observed in the CYP (*p* < 0.001) and R groups (*p* < 0.05, *p* < 0.001, respectively).

The CYP group exhibited a substantial increase in ALT and AST, with levels rising by approximately 73.6% and 66.3%, respectively, compared to the control group (*p* < 0.001). Similarly, the R group displayed elevated ALT and AST levels, showing an increase of about 45.4% (*p* < 0.05) and 57.9% (*p* < 0.001), respectively, relative to controls, indicating significant hepatotoxicity induced by these treatments. The combination of CYP and R (CYP + R group) resulted in the most pronounced changes, with ALT increasing by approximately 129% (*p* < 0.001) and AST by approximately 102% (*p* < 0.001) compared to the control group, suggesting a synergistic effect of CYP and R on liver damage.

Interestingly, the inclusion of STS with either CYP or R appeared to attenuate the increase in liver enzyme levels. The STS + CYP group showed increases of approximately 35.9% and 32% in ALT and AST, respectively, relative to the control (*p* > 0.05), with AST being significantly lower than in the CYP group alone (*p* > 0.05). Similarly, the STS + R group displayed reduced ALT and AST levels, with increases of only about 10.2% and 19.1%, respectively, compared to the control group (*p* > 0.05), with significant reductions in AST compared to both R (*p* < 0.05) and CYP groups (*p* ≤ 0.01). However, when STS was combined with both CYP and R (STS + CYP + R group), the percentage changes in ALT and AST were higher than those in the STS + CYP or STS + R groups, with increases of approximately 63.7% (*p* < 0.01) and 55.9% (*p* < 0.001), respectively, which were significantly higher than those in the control group but significantly lower than in the CYP + R group alone (*p* ≤ 0.001).

These results suggest that CYP and R treatments induce significant hepatotoxicity, as evidenced by the large percentage increases in ALT and AST levels. STS appears to offer some protection by attenuating these elevations when combined with either treatment individually or together, although it does not completely prevent the damage caused by CYP + R (Table 2).

**Table 2:**
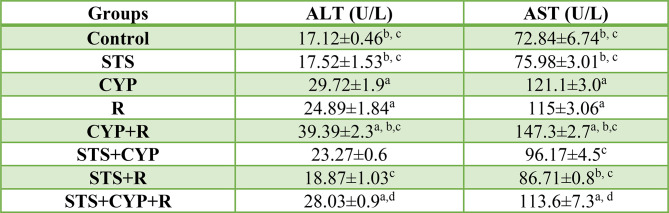
The protective effect of STS against CYP and/or R-induced changes in serum levels of liver enzymes

Data were expressed as mean ± SEM. Values were statistically significant at *p* ≤ 0.05. ^a^: Significant compared to the control group; ^b^: Significant compared to the R group, ^c^: Significant compared to the CYP group, and ^d^: Significant compared to the CYP + R group. Statistical significance was determined using one-way ANOVA followed by Tukey’s post-hoc test at *p* ≤ 0.05 for all comparisons. One international unit (IU) is the amount of enzyme that transforms 1 µmol of substrate per minute

### Effect of STS on pancreatic and hepatic oxidative stress biomarkers

**Fig. 2:**
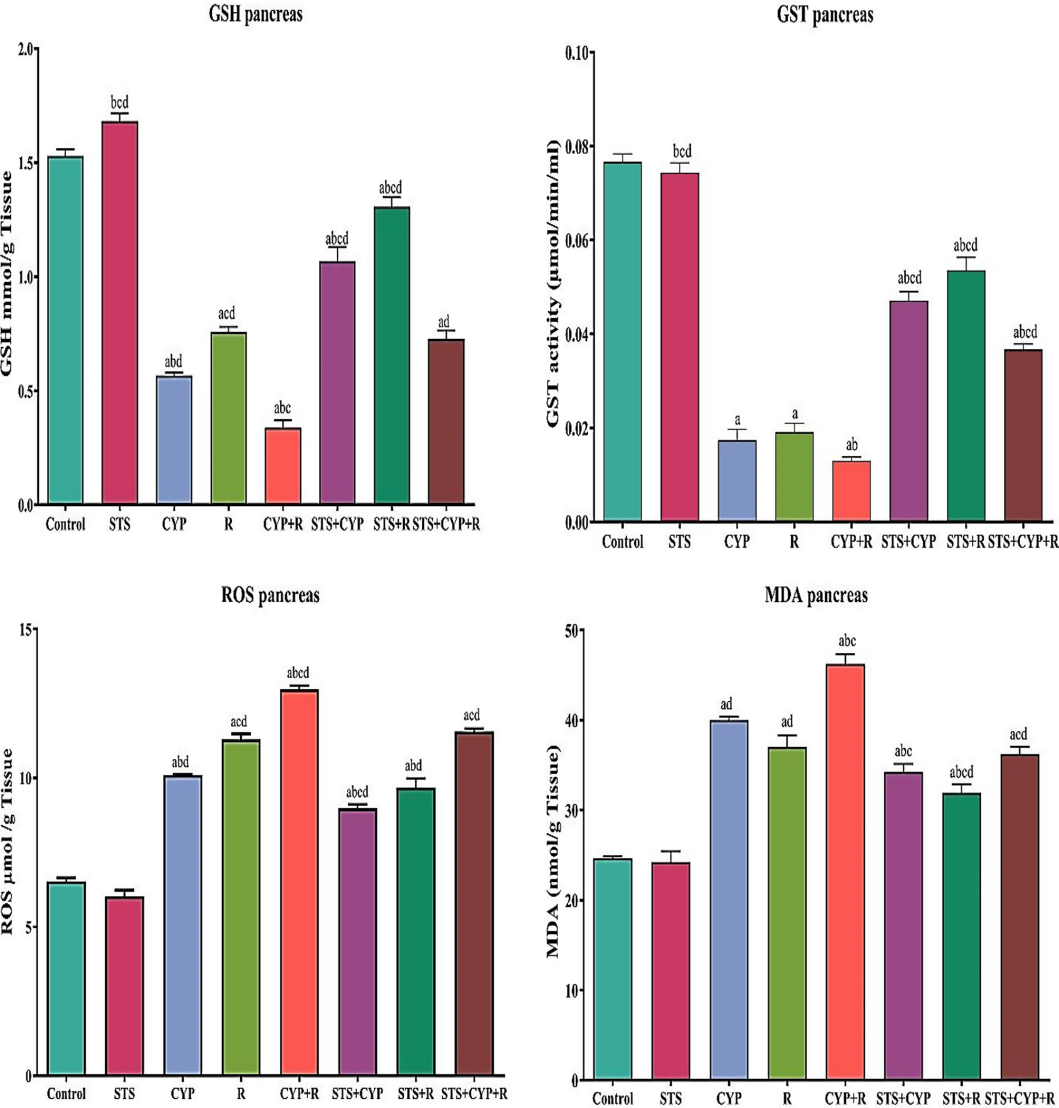
Effect of STS treatment on the pancreatic levels of GSH, GST, ROS, and MDA in male rats in all experimental groups. Data were expressed as mean ± SEM. Values were statistically significant at *p* ≤ 0.05. ^a^: Significant compared to the control group, ^b^: Significant compared to the R group, ^c^: Significant compared to the CYP group, and ^d^: Significant compared to the CYP + R group. Statistical significance was determined using one-way ANOVA followed by Tukey’s post-hoc test at *p* ≤ 0.05 for all comparisons

**Fig. 3:**
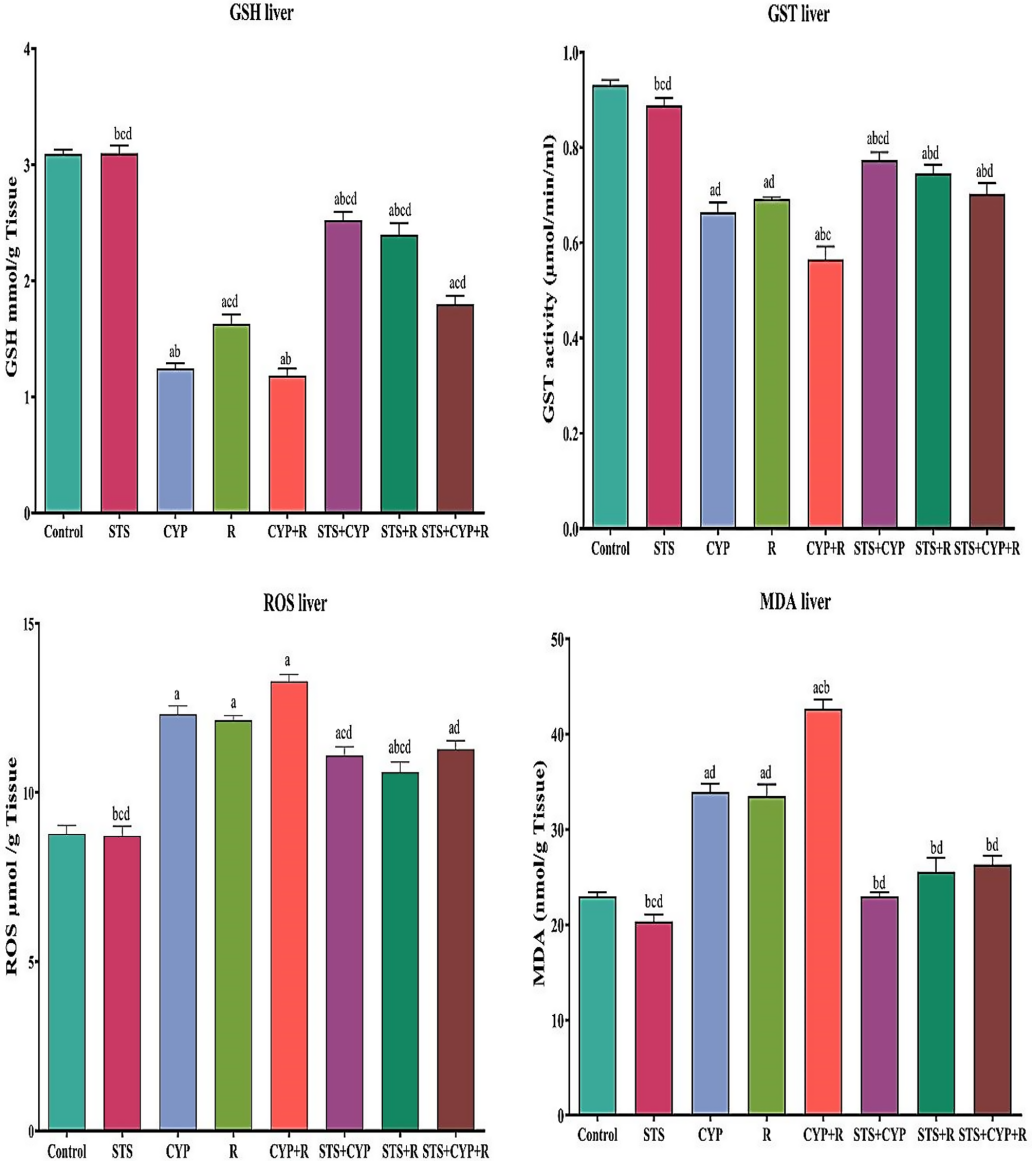
Effect of STS treatment on the hepatic levels of GSH, GST, ROS, and MDA in male rats in all experimental groups. Data were expressed as mean ± SEM. Values were statistically significant at *p* ≤ 0.05. ^a^: Significant compared to the control group, ^b^: Significant compared to the R group, ^c^: Significant compared to the CYP group, and ^d^: Significant compared to the CYP + R group. Statistical significance was determined using one-way ANOVA followed by Tukey’s post-hoc test at *p* ≤ 0.05 for all comparisons

An imbalance in the oxidative status of pancreatic and hepatic tissues was brought on by the administration of CYP and/or R to rats. As seen in Figs. (2 & 3), control, CYP and/or R groups displayed an increase in MDA and ROS levels as well as a decrease in GSH and GST activities. Contrarily, STS attitudes significantly increased (*p* < 0.001) the pancreatic and hepatic activities of GSH and GST, decreased MDA and ROS levels, and significantly reversed (*p* < 0.001) the redox imbalance demonstrated by CYP and/or R as compared to the untreated groups. An insignificant difference (*P* > 0.05) between the STS group and the control group was also noticed.

### Comparative analysis of serum glucose, insulin, amylase, and lipase levels in control and experimental rats

**Fig. 4:**
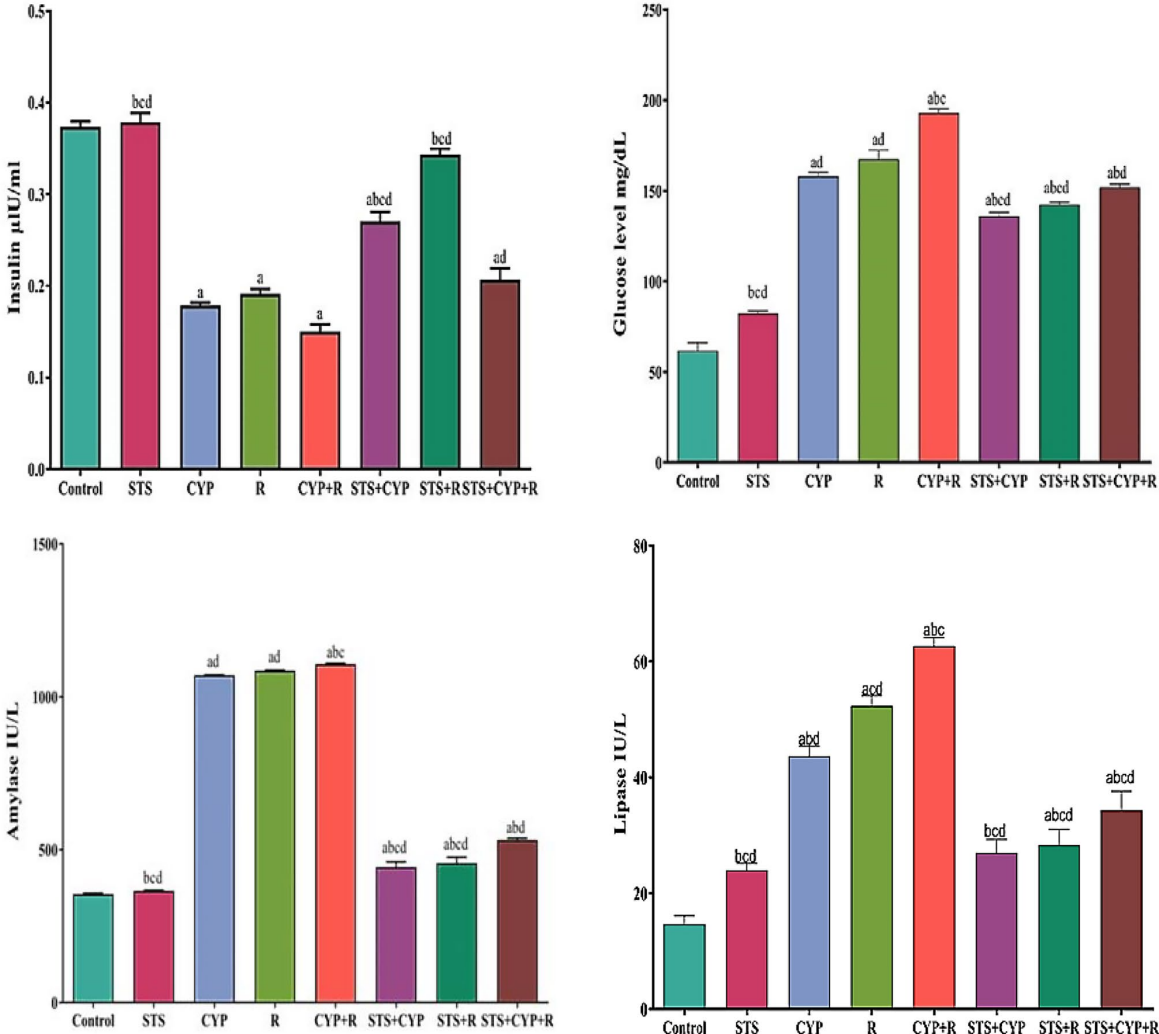
Effect of STS on serum insulin, glucose, amylase, and lipase activities in male rats across all experimental groups. Data were expressed as mean ± SEM. Values were statistically significant at *p* ≤ 0.05. ^a^: Significant compared to the control group, ^b^: Significant compared to the R group, ^c^: Significant compared to the CYP group, and ^d^: Significant compared to the CYP + R group. Statistical significance was determined using one-way ANOVA followed by Tukey’s post-hoc test at *p* ≤ 0.05 for all comparisons

In the present study, as represented in Fig. 4, serum glucose, lipase, and amylase levels were higher in the CYP and/or R-treated rats (CYP, R, and CYP + R groups), and the results were significant (*P* < 0.001), indicating both endocrine and exocrine cell damage. On the other hand, oral administration with STS (STS + CYP, STS + R, and STS + CYP + R groups) produced significant suppression of serum glucose (13.9, 14.9, and 21.2%), amylase (56.5, 48.6, and 45.9%), and lipase (38.1, 41.08, and 41.2%) activities as compared to the untreated groups, respectively. Moreover, there was a significant decrease in insulin hormone levels (*P* < 0.001) in the CYP, R, and CYP + R groups by 52.2, 48.7, and 59.8%, respectively, as compared to the control group. Meanwhile, STS showed an ameliorating effect on the insulin level by 33.9, 44.2, and 27.3%, respectively, compared to untreated groups.

### Effect of STS on gene expression of Erk-1 and Jnk

**Fig. 5:**
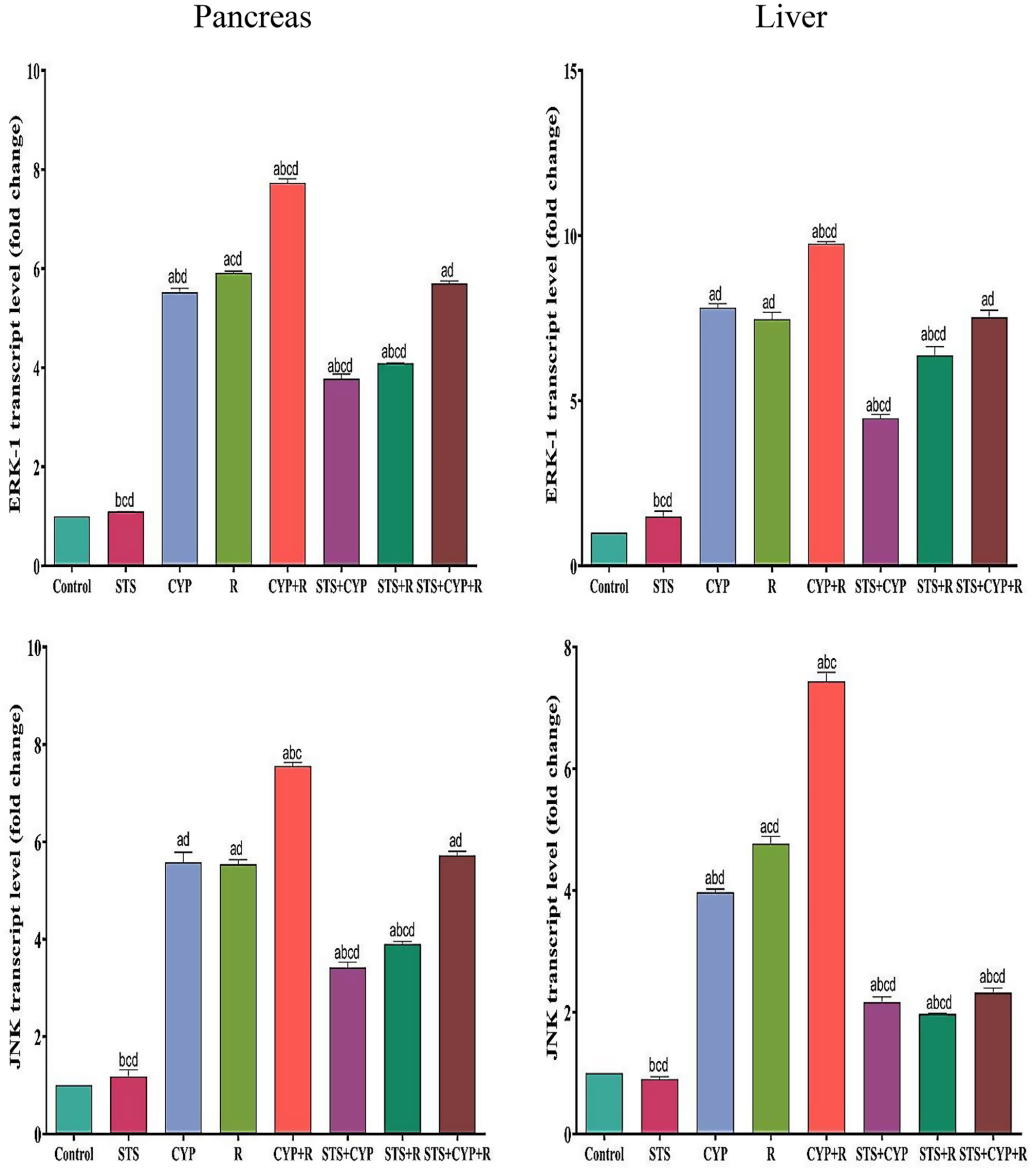
Effect of STS on both pancreatic (left side) and hepatic (right side) levels of ERK and JNK in male rats across all experimental groups. Data were expressed as mean ± SEM. Values were statistically significant at *p* ≤ 0.05. ^a^: Significant compared to the control group, ^b^: Significant compared to the R group, ^c^: Significant compared to the CYP group, and ^d^: Significant compared to the CYP + R group. Statistical significance was determined using one-way ANOVA followed by Tukey’s post-hoc test at *p* ≤ 0.05 for all comparisons

The transcript levels of ERK-1 and JNK were evaluated in both the liver and pancreatic tissues across all experimental groups, as depicted in Fig. 5. In the control group, the mean ERK-1 expression level was 1.0 ± 0.0 in both liver and pancreas. Treatment with STS alone resulted in a slight increase in ERK-1 expression in both tissues (liver: 1.49 ± 0.16, pancreas: 1.09 ± 0.003). Treatment with CYP alone or R alone significantly increased ERK-1 expression in both the liver (7.823 ± 0.11) and pancreas (5.52 ± 0.08 for CYP, 5.9 ± 0.03 for IR). The combination of CYP and R exhibited the highest increase in ERK-1 expression in both liver (9.760 ± 0.06) and pancreas (7.73 ± 0.08). Co-administration of STS with CYP or R reduced ERK-1 expression compared to the CYP and R alone groups in both tissues. Furthermore, the combination of STS, CYP, and R showed lower ERK-1 expression compared to the CYP and R alone groups in both liver (7.53 ± 0.21) and pancreas (5.70 ± 0.05). Regarding JNK expression, in the control group, the mean level was 1.0 ± 0.0 in both liver and pancreas. Treatment with STS alone led to a decrease in JNK expression in both tissues (liver: 0.90 ± 0.039, pancreas: 1.18 ± 0.13). Treatment with CYP alone or R alone increased JNK expression in both liver (3.97 ± 0.05 for CYP, 4.7 ± 0.12 for IR) and pancreas (5.58 ± 0.20 for CYP, 5.5 ± 0.09 for IR). The combination of CYP and R exhibited the highest increase in JNK expression in both liver (7.43 ± 0.14) and pancreas (7.55 ± 0.07). Co-administration of STS with CYP or R reduced JNK expression compared to the CYP and R alone groups in both tissues. Moreover, the combination of STS, CYP, and R displayed lower JNK expression compared to the CYP and R alone groups in both liver (2.32 ± 0.07) and pancreas (5.72 ± 0.08). The treatments involving CYP, R, and STS exhibited distinct effects on the expression levels of ERK-1 and JNK, suggesting potential interactions and modulations in these signaling pathways in both the liver and pancreas.

### Histopathological findings of STS effect on CYP and/or R-induced pancreatic damage in male rats

**Fig. 6:**
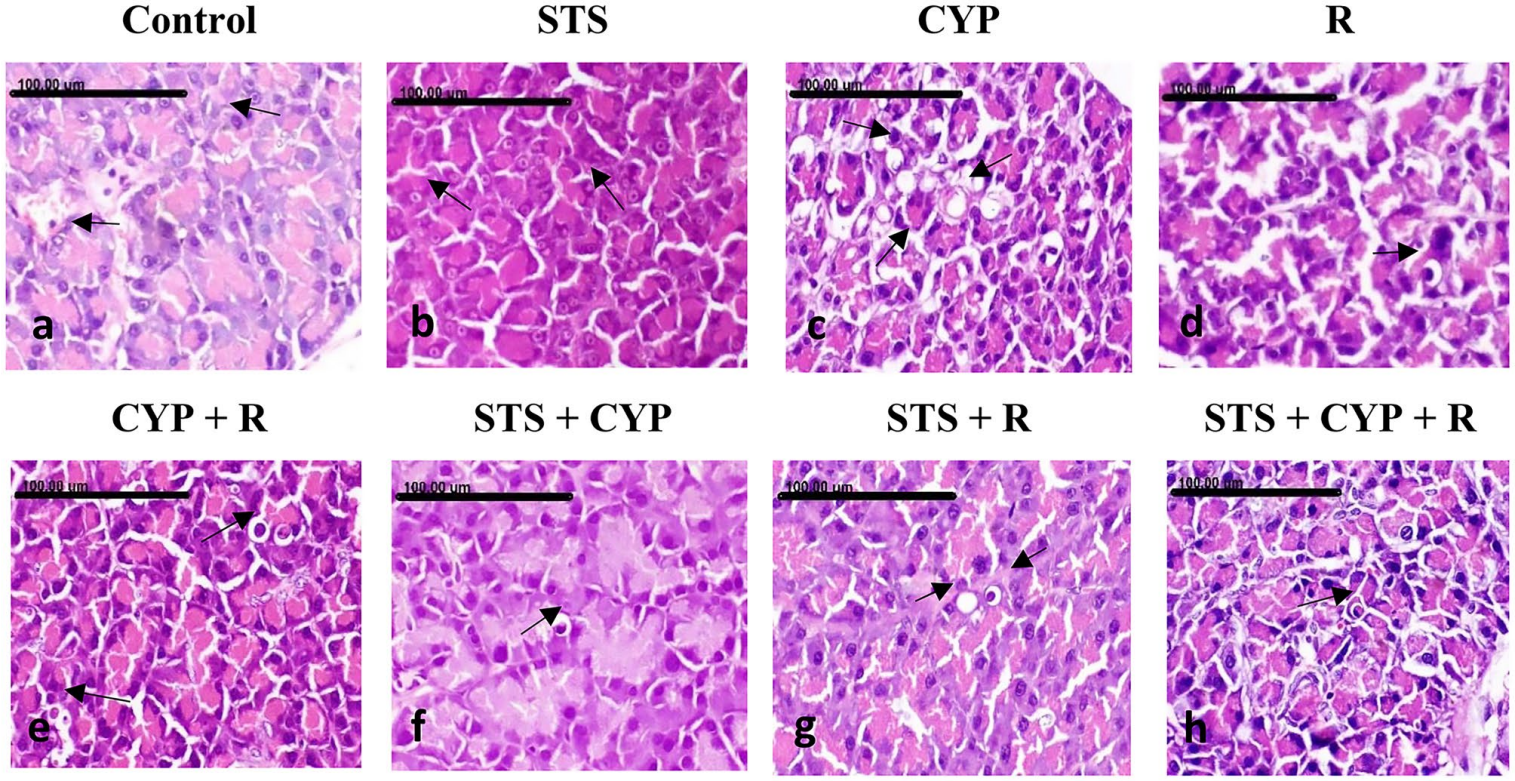
Photomicrograph of exocrine pancreatic tissue sections showing (**a**, **b**): normal pancreatic acinar cells (arrows) and (**c**) showing vacuolar degeneration of the exocrine acinar cells in the CYP group (arrow) (**d**) few numbers of apoptotic bodies in the exocrine acinar cells of R group (arrows), (**e**) numerous apoptotic bodies in CYP + R group (arrows) (**f**) individual apoptotic body in STS + CYP group (black arrows), (**g**) vacuolization of acinar cells in STS + R group (arrow) and sparse apoptotic body (arrows) (**h**) abundant apoptotic bodies in STS + CYP + R group (black arrows). (Stain: H&E, x400. Scale bar = 100 μm)

**Fig. 7:**
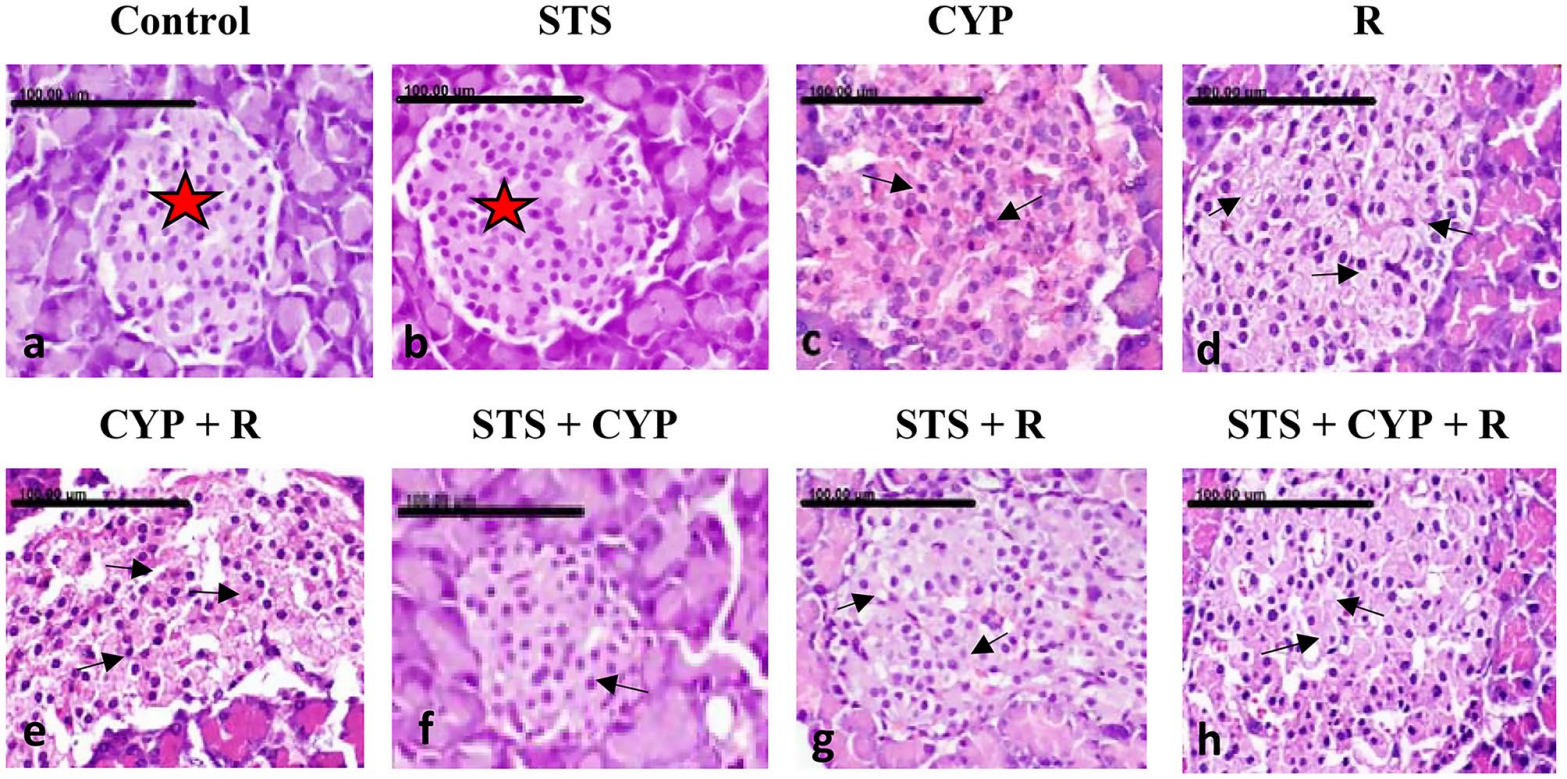
Photomicrograph of endocrine pancreatic tissue sections showing: (**a**, **b**) normal Islet of Langerhans (Asterix) (**c**) numerous apoptotic cells in the CYP group (arrow) (**d**) necrosis of the Islet of Langerhans in the R group (arrows) (**e**) multifocal necrosis in CYP + R group (arrow) of Langerhans cells (**f**) individual apoptotic bodies in the islets of Langerhans in the STS + CYP group (arrow) (**g**) few numbers of apoptotic islet cells in the STS + R group (arrow) (**h**) frequent single-cell necrosis in the islets of Langerhans in STS + CYP + R group (black arrows). (Stain: H&E, x400.Scale bar = 100 μm)

Normal histological structure of exocrine and endocrine pancreatic acinar cells without evidence of necrosis and/or apoptosis was demonstrated in the control animals group (Figs. 6a and 7a) and the STS group (Figs. 6b and 7b). On the contrary, vacuolar degeneration of exocrine acinar cells, with numerous small and large vacuoles, was seen in the CYP group (Fig. 6c). In addition, numerous apoptotic cells were noticed in the islet of Langerhans. The apoptotic cells appeared shrunken, with intensely eosinophilic cytoplasm and condensed nucleolar chromatin (Fig. 7c). Similarly, abundant apoptotic bodies were found in the exocrine pancreatic acinar cells of the R group (Fig. 6d). The necrotic cells appeared swollen, with pale eosinophilic cytoplasm with nuclear pyknosis (Fig. 7d). More severe pathological alterations with numerous numbers of apoptotic bodies were demonstrated in the exocrine acinar cells of the CYP + R group (Fig. 6e). Apoptosis of the Langerhans cells was also displayed (Fig. 7e). Pronounced improvement of the pathological alterations was demonstrated in the pancreatic tissue sections of the STS + CYP and the STS + R groups. Sparse apoptotic bodies were demonstrated in the exocrine acinar cells of the STS + CYP and the STS + R groups (Fig. 6f and g). The islets of Langerhans in the STS + CYP group showed a few numbers of apoptotic bodies (Fig. 7f). Restoration of Langerhans islets with decreased numbers of apoptotic cells was seen in the STS + R group (Fig. 7g). Mild amelioration was recorded in the pancreas of the STS + CYP + R group, which revealed abundant apoptotic bodies in the exocrine acinar cells and Langerhans islets cells (Figs. 6h and 7h).

### Effect of STS on hepatocellular injury induced by CYP and/or R in male rats

**Fig. 8:**
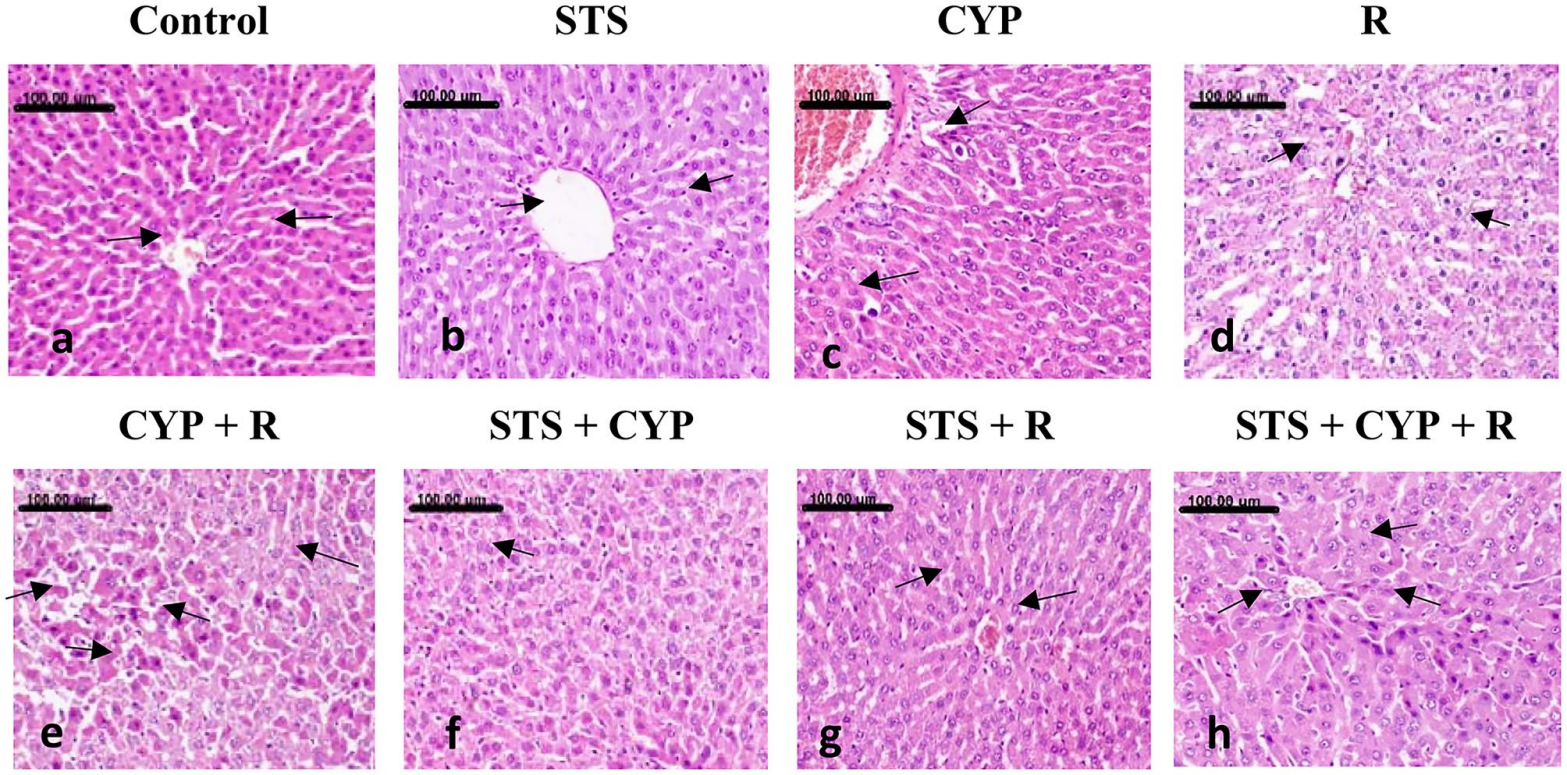
Showing the hepatic tissues of the different groups: (**a**) normal control group showing normal hepatocytes with round vesicular nuclei (arrows), (**b**) STS group showing normal hepatocytes with normal nuclei (arrow), (**c**) CYP group showing increased number of apoptotic bodies (arrows), (**d**) R group showing extensive vacuolar degeneration of hepatocytes, which appeared markedly swollen, with reticulation of their cytoplasm (arrows), (**e**) CYP + R group showing diffuse vacuolar degeneration of hepatocytes (arrows) alternating with extensive necrosis and/or apoptosis of hepatocytes (black arrows), (**f**) STS + CYP group showing individual apoptotic body (arrow) (**g**) STS + R group showing marked regression of hepatocellular swelling (arrows), and (**h**) STS + CYP + R group showing sporadic cell necrosis (arrows) (Stain: H&E, x200.Scale bar = 100 μm)

**Fig. 9:**
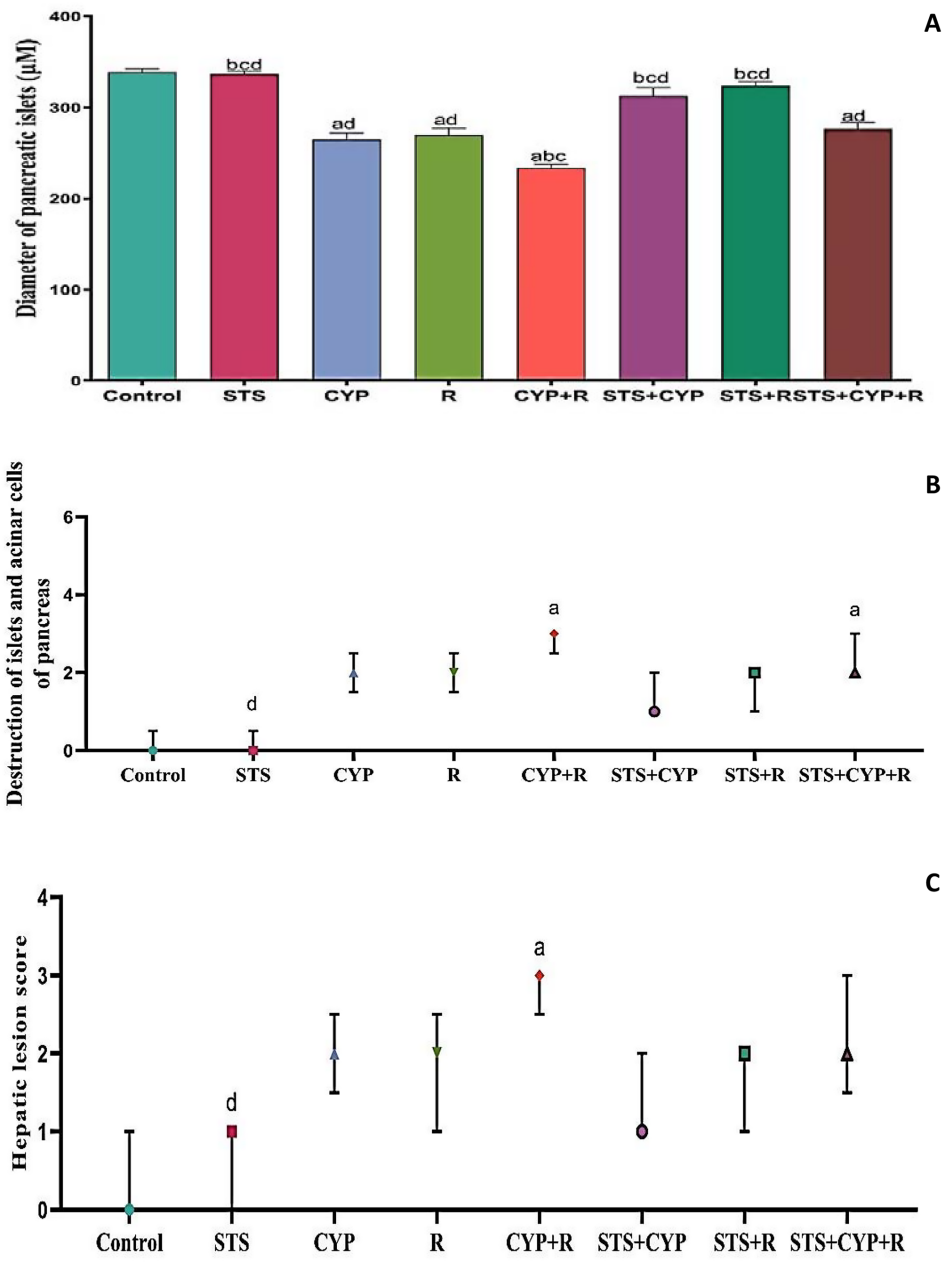
Effect of STS on pancreatic and hepatic lesion score in CYP and/or R-induced pancreatic injury and liver toxicity; (**A**) Diameter of pancreatic islets presented as mean ± SEM analyzed using one-way ANOVA. (**B**) Destruction of islet cells and c) Hepatic lesion score. (**B**) and (**C**) were presented as median with an interquartile range (IQR; *n* = 8) and analyzed using the Kruskal-Wallis’s test followed by Dunn’s Multiple Comparison Test. ^a^: Significant compared to the control group,^b^: Significant compared to the R group, ^c^: Significant compared to the CYP group, and ^d^: Significant compared to the CYP + R group

Normal hepatic architecture with normally arranged hepatocytes was demonstrated in the livers of the normal control and STS groups (Fig. 8a and b). In contrast, pronounced hepatocellular damage was demonstrated in the livers of the CYP and R groups, as numerous numbers of apoptotic cells and extensive vacuolar degeneration of hepatocytes appeared (Fig. 8c and d). More severe hepatocellular damage was demonstrated in the CYP + R group, which revealed diffuse vacuolar degeneration of hepatocytes with extensive necrosis and/or apoptosis of hepatocytes (Fig. 8e). Significant amelioration of hepatic lesions was noticed in the STS + CYP and STS + R groups. Mild granular degeneration of hepatocytes associated with a few number of apoptotic bodies was demonstrated in the STS + CYP group (Fig. 8f). A marked regression of hepatocellular swelling was seen in STS + R group (Fig. 8g). In comparison to STS + CYP and the STS + R groups, mild improvement was displayed in STS + CYP + R group which revealed sporadic apoptotic cells (Fig. 8h). The pathologic lesions scoring for pancreatic and hepatic injuries were illustrated in (Fig. 9).

### Immunohistochemical evaluation of pancreatic and hepatic Nrf2 expression against different experimental groups

**Fig. 10:**
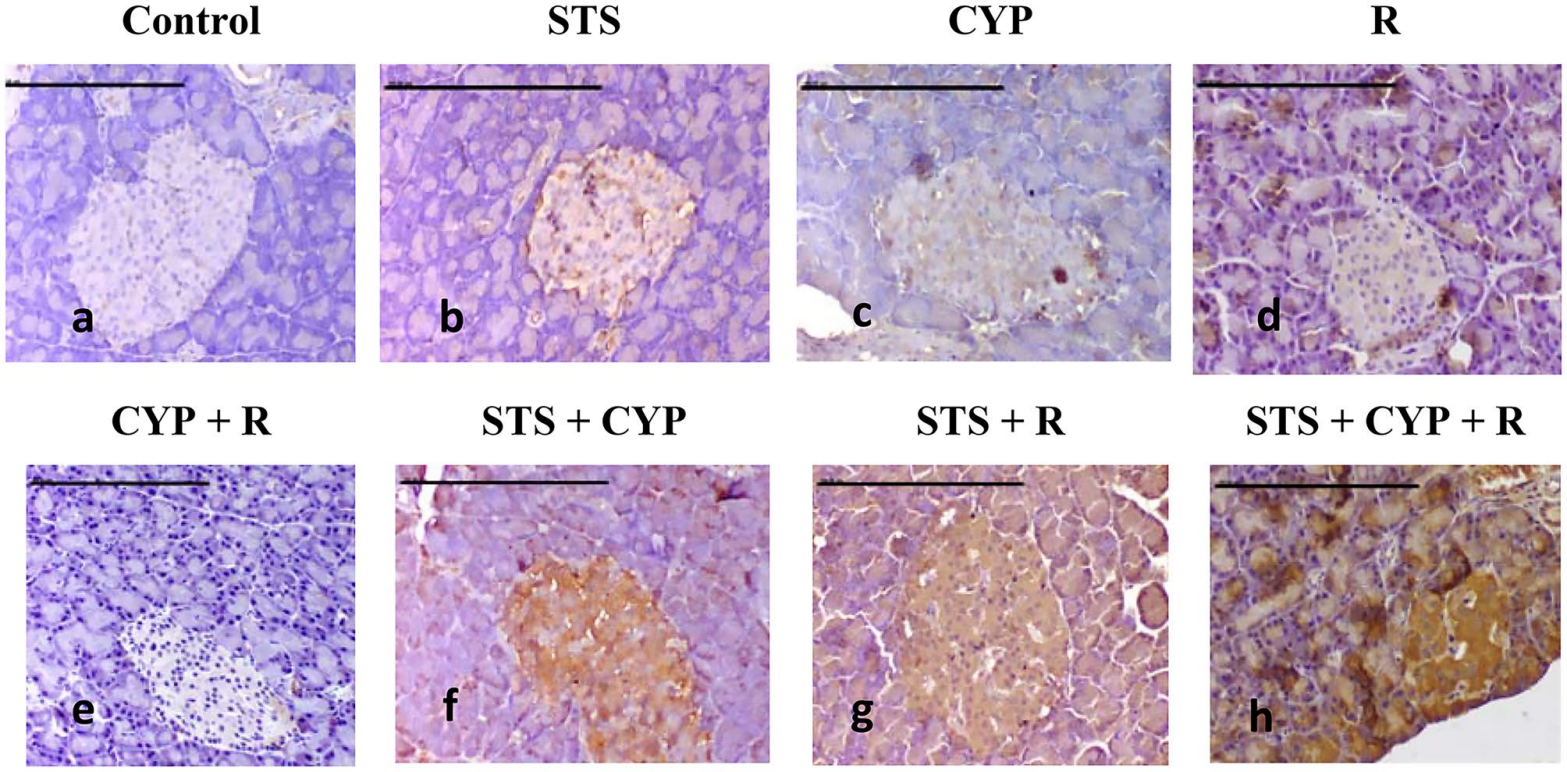
Photomicrograph of pancreatic immunohistochemical Nrf2 expression: (**a**&**b**) Mild nuclear Nrf2 expression was demonstrated in islet and acinar cells of control and STS groups (**c**), weak brown staining of the cytoplasm and/or nuclei of the pancreatic tissue (**d**), less Nrf2 expression of pancreas (**e**), absence of positively stained cells with no brown staining of islet and acinar cells (**f**), and (**g**) increase of Nrf2 expression of pancreatic tissue (**h**), strong cytoplasmic and/or nuclear staining of endocrine and exocrine parts (scale bar = 100 μm)

**Fig. 11:**
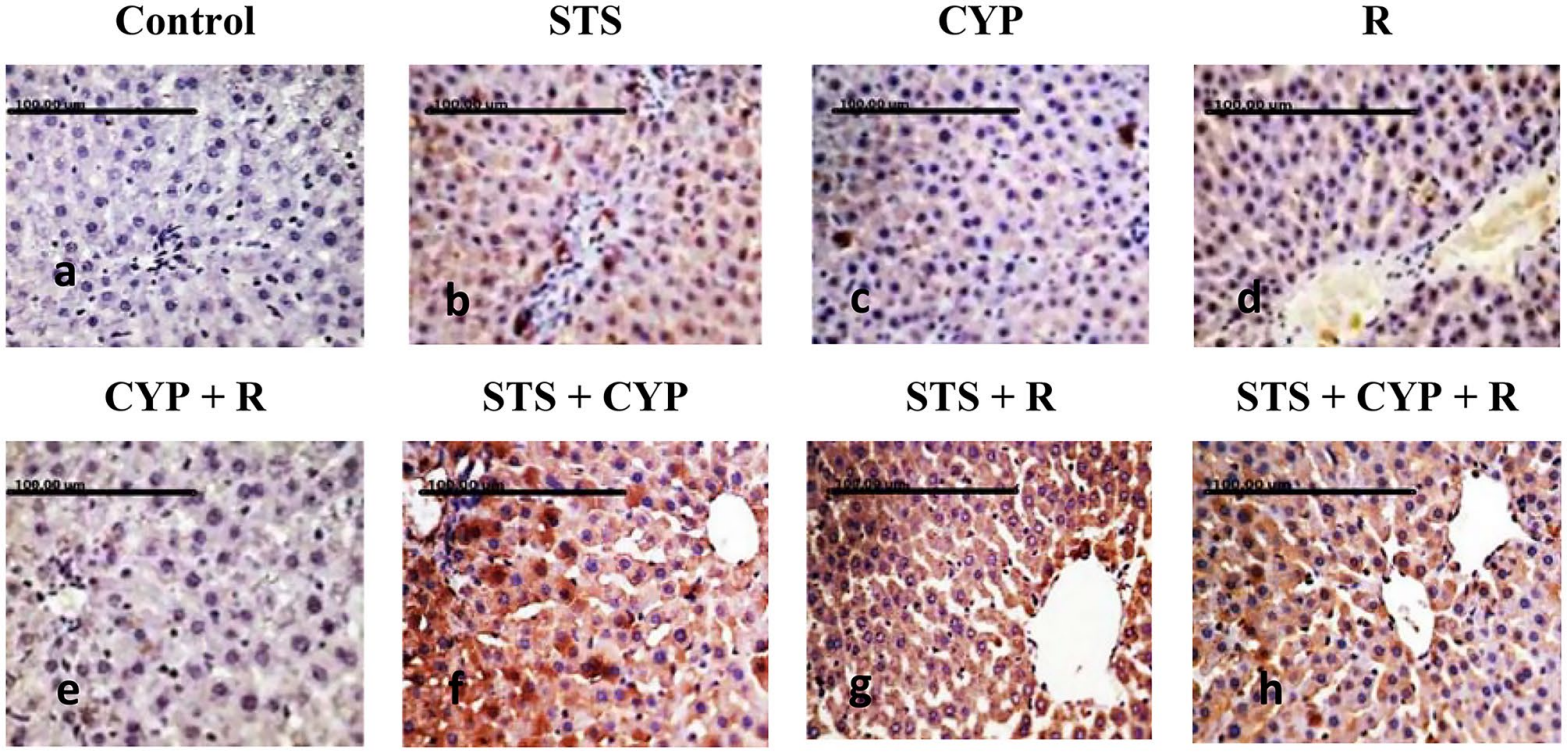
Photomicrograph of hepatic immunohistochemical Nrf2 expression: (**a**&**b**) Mild Nrf2 expression was demonstrated in hepatocytes of the control and STS groups, (**c**) weak brown staining of the cytoplasm and/or nuclei of hepatocytes, (**d**) mild positive Nrf2 expression of hepatic tissue, (**e**) there are no positively stained cells and no brown staining of hepatocytes, (**f**) & (**g**) expression of Nrf2 increases with strong brown staining, (**h**) most hepatic tissues show strong cytoplasmic and nuclear staining (scale ba *r* = 100 μm)

Mild expression of Nrf2 was demonstrated in the pancreas of the normal control group (Fig. 10a), and more Nrf2 expression was observed in the STS group (*p* > 0.05) (Fig. 10b). In contrast, Nrf2 expression was reduced in islet and acinar cells of the CYP group (*p* > 0.05) (Fig. 10c). Weekly positive immune-stained cells were recorded in the R group (*p* > 0.05) (Fig. 10d). A non-significant decrease (*p* > 0.05) of Nrf2-positive cells was recorded in the exocrine and endocrine pancreatic tissues of the CYP + R group compared to CYP or R groups (Fig. 10e). On the other hand, pretreatment with STS revealed a significant increase in Nrf2 expression, particularly in the pancreatic tissue sections of STS + CYP (*p* < 0.001) and STS + R (*p* < 0.01) (Fig. 10f and g). Additionally, Nrf2-strong positively stained cells were demonstrated in the STS + CYP + R group (Fig. 10h). Control and STS groups showed moderate immunostaining affinity for Nrf2 (*p* > 0.05) (Figs. 11a & b).

**Fig. 12:**
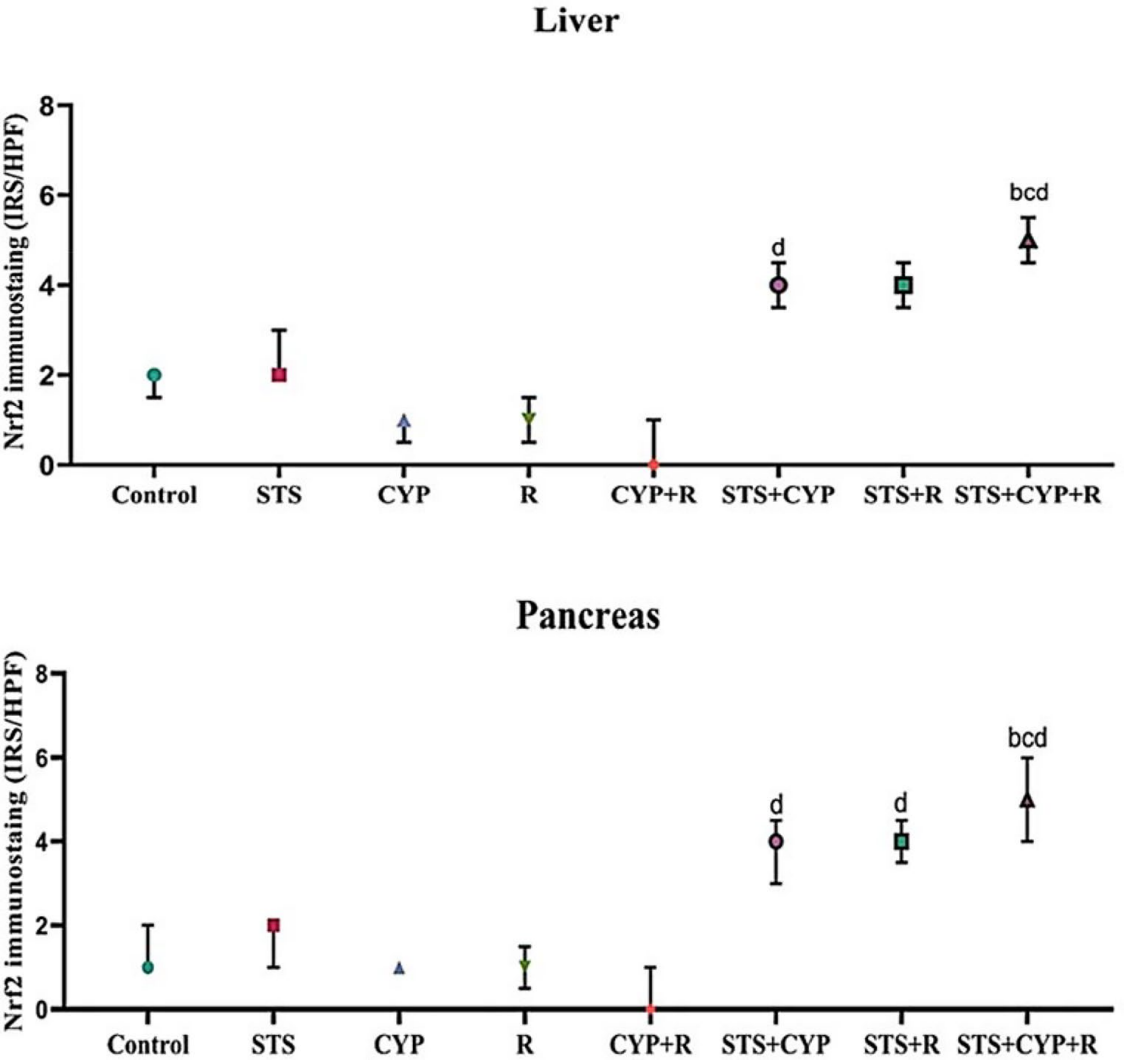
Pancreatic and hepatic Nrf2 immunohistochemical scoring. All results were presented as medians with an interquartile range (IQ; *n* = 8), by Kruskal Wallis H test. ^a^: Significant compared to the control group, ^b^: Significant compared to the R group, ^c^: Significant compared to the CYP group and ^d^: Significant compared to the CYP + R group. (Kruskal–Wallis test, Dunn’s correction for multiple testing at *p* < 0.05)

CYP administration and/or R exposure revealed a decrease in Nrf2-positive hepatocytes, which was evident by weak positive brown staining (*p* > 0.05) compared to control. The expression of Nrf2 in hepatocytes was mainly detected at nuclear and cytoplasmic levels in the CYP group (Fig. 11c) and R group (Fig. 11d). A more conspicuous decrease of Nrf2 positively stained cells, with strong brown staining, was demonstrated in hepatocytes of the CYP + R group (*p* < 0.001) (Fig. 11e). In contrast, in STS-treated groups, the hepatic tissue sections showed slight stain affinity for Nrf2 expression in comparison to CYP or R groups (Fig. 11f and g). However, strong expression was recorded in the STS + CYP + R group, which revealed relatively abundant nuclear and cytoplasmic positively brown staining in hepatic tissue (*p* < 0.001) (Fig. 11h). The total immune reactivity score (TIS) of Nrf2 recorded in the pancreas and liver of different animal groups was illustrated (Fig. 12).

## Discussion

Radiotherapy and chemotherapeutic treatment cause unwanted side effects [49, 50]. Reactive radicals and oxidants have the potential to damage cells and tissues [50]. Oxidative stress induced by CYP and/or R leads to pancreatic damage and hepatotoxicity [51, 52]. In the current study, CYP and/or R revealed a significant increase in serum AST and ALT levels [53, 54]. Moreover, CYP has a close relation to the increase of alkaline phosphatase and AST levels [55]. The CYP and/or R, are toxic not only to cancer cells but also to healthy cells, particularly those with a high rate of proliferation, which may result in serious side effects for patients [56, 57]. Exposure to the CYP and/or R administration produced significant alterations in the antioxidant activity in different tissues [56, 57]. In the present study, ROS and MDA levels were increased in CYP and/or R groups due to the effect of free radicals on the fatty acid component of membrane lipids [58]. MDA is a cellular polyunsaturated fatty acid marker of oxidative stress that is produced [59]. The antioxidant enzymes capable of scavenging ROS are GSH and GST [60]. GSH plays a vital role in protecting cells against oxidative injury. GSH depletion caused by CYP and/or R treatment reduces its cellular level, resulting in the induction of oxidative stress [61]. In this study, we observed a decrease in the activities of GSH and GST in CYP and/or IR-treated pancreas and hepatocytes. This decrease could be due to a feedback inhibition or oxidative inactivation of enzyme proteins caused by ROS generation, which in turn can impair the antioxidant defense mechanism, leading to increased membrane lipid peroxidation (LPO) [62, 63]. Pretreatment with STS significantly reduced the serum levels of ALT and AST. The protective effect of STS is related to its antioxidant activity through the direct inhibition of ROS, adjustment of GSH cellular levels, and increased expression of antioxidant enzymes [64]. Besides its antioxidant activity, STS has been shown to have anti-inflammatory properties by decreasing the formation of IL-1β and other pro-inflammatory cytokines [65]. To investigate the effect of STS on CYP and/or IR, the serum level of amylase was measured. The results mentioned in Fig. 4 indicated that at the end of treatment, CYP and/or R significantly increased the serum level of amylase as compared to that of the control group. This indicated the disruption of normal pancreatic function by CYP and/or R, due to which the production of the carbohydrate metabolizing enzyme (amylase) was increased, which in turn might have increased the production of glucose. Amylase and lipase levels in the current study were significantly lower in the STS-treated group. These findings were agreed with [66], who proposed that H_2_S pretreatment was associated with a decreased incidence of pancreatitis. Moreover, pretreatment with H_2_S was accompanied by a reduction of proinflammatory chemokines, inhibition of reactive oxygen species, and increased expression of antioxidant enzymes. The effect of STS on fat metabolism by measuring the serum level of lipase was also assessed. Lipase is an enzyme that plays an active role in hydrolyzing lipids and ester bonds in triglycerides to form fatty acids and triglycerides. At the end of treatment, we found that STS significantly decreased the serum level of the lipase enzyme in the CYP and/or R groups as compared to the control one [67]. Pancreatic acinar cells are severely affected, with elevation of serum amylase and lipase levels in CYP and/or R groups confirming this injury and being attributed to necrosis and/or apoptosis of pancreatic acinar cells [68]. The current investigations found a significant decrease in serum insulin and an increase in glucose levels. These biochemical changes are confirmed by the results of histopathology, which revealed significant changes in the endocrine pancreas, as evidenced by numerous apoptosis of β-cell of Langerhans islet cells, especially in the combination group CYP + R, resulting in endocrine insufficiency. This endocrine insufficiency explains the significant decrease in insulin levels in CYP and/or R-treated rats [69]. Furthermore, the elevation of glucose level was recorded in CYP and/or R groups due to widespread β-cell destruction, as confirmed by [70, 71], who reported hyperglycemia following CYP and/or R therapy. On the other hand, high insulin levels and low serum glucose levels were detected in STS + CYP, STS + R and STS + CYP + R groups. Treatment with STS protects pancreatic cells in three ways, including a reduction in ROS production, inhibiting the expression of thioredoxin-binding protein 2, a redox protein associated with diabetes that promotes apoptosis, and increasing GSH content, all of which reduce the damage caused by oxidative stress [72–74]. Administration of STS can increase glucose production by inhibiting the serine/threonine kinase family Akt and activating phosphoenolpyruvate carboxykinase, according to Mani et al. [75]. Moreover, insulin secretion is usually affected by many factors. It is known that the concentration and oscillation of Ca^2+^ and K_ATP_ channels are related to H_2_S. Treatment with H_2_S not only inhibits the entry of Ca^2+^ from the plasma membrane into cells to reduce insulin secretion but also promotes the release of Ca^2+^ in mitochondria and increases insulin secretion [76]. Necrosis and/or apoptosis of exocrine pancreatic tissues in the CYP + R group may result from increased ROS and MDA concentrations, as well as lower GSH and GST concentrations and increased levels of pro-inflammatory markers such as TNF-α [58]. Moreover, the pancreatic acinar cells are much more sensitive to R damage than islet cells, as reported by Kleinerman et al. [70]. This study also revealed a significant upregulation of ERK-1 and JNK in the pancreas and liver. This is consistent with the findings of Kyriakis & Avruch [77], who found that CYP activation of MAPK superfamily ERK and JNK signaling pathways involves a family of serine/threonine protein kinases that regulate cell proliferation, cell survival, and apoptosis. JNK and ERK play critical roles in the induction of apoptosis. JNK and ERK, along with p38, are primarily involved in the response to extracellular and intracellular stress, with subsequent JNK and ERK activating the pro-apoptotic Bcl-2 protein [78]. Furthermore, ROS overproduction after CYP administration induces JNK and ERK expression, which in turn induces apoptosis via increased expression of TNF-α, caspase-8, and caspase-3 [77]. Not only CYP but also R regulates the MAPKs (ERK and JNK) that are thought to be essential in cell survival or death. Sheen and Dickson [79] demonstrated that R generates ROS, which is associated by increased cell proliferation and transcription of the MAPK/ERK/JNK signaling pathways. Pretreatment with STS is usually accompanied by a decrease in MAPK expression, resulting in the suppression of oxidative stress-mediated activation of TLR4/MAPK/p38/NF-B signaling pathways [80]. H_2_S has anti-apoptotic, antioxidant and anti-inflammatory properties, which are reflected in the histological structures of the pancreas and liver. Immunohistochemical analysis revealed a decrease in pancreatic and hepatic Nrf2 expression. Nrf2 is a transcription factor that induces the expression of various cytoprotective enzymes [81]. It has been reported that CYP and/or R reduce the nuclear level of Nrf2 [82, 83]. The Nrf2-mediated adaptive response attenuates toxicity and carcinogenesis during exposure to electrophiles or oxidative stress in rats [81]. Nrf2 acts as a regulator of cellular antioxidant activity and the detoxifying defense system. Dysregulation of Nrf2 expression is correlated with a disturbance in pancreatic and hepatic function and the development of oxidative stress [84]. In the present study, Nrf2 dysregulation was associated with the deactivation of the antioxidant enzymes GSH and GST as well as an increase in ROS levels. The depletion of the antioxidant defense system reflects the severity of pathological alterations in both the pancreas and liver [85, 86]. Our findings agreed with previous studies [87, 88]; they suggested that these findings could be mediated by CYP and/or IR, which induce ROS overproduction, resulting in the inhibition of Nrf2 and its related cellular defense enzymes. In the current study, STS supplementation maintained and repaired pancreatic and hepatic Nrf2 homeostasis and induced its related detoxifying and antioxidant molecules. This could indicate that STS protects against CYP and/or R-mediated oxidative reactions [65]. These findings could be attributed to the ability of STS to scavenge ROS as well as the fact that STS has an antioxidant effect via stimulation of several antioxidants such as nuclear Nrf2 and modulation of various pro-inflammatory signal mechanisms such as NF-κβ and attenuated TNF-α [89, 90].

We acknowledge the limitation of not analyzing apoptotic proteins; however, the identification of apoptotic cells through H-E staining provides valuable morphological evidence. Existing literature supports the relationship between JNK/ERK signaling and apoptosis, with studies indicating that JNK activation promotes apoptosis, while ERK can have context-dependent roles in cell survival or apoptosis [91, 92]. These findings reinforce the relevance of our results, as STS-mediated modulation of these pathways may influence apoptotic processes. Future studies are recommended to directly analyze apoptotic proteins and further elucidate the mechanisms underlying STS’s protective effects on liver and pancreatic function.

## Conclusion

It was concluded that the present study establishes STS as an effective protective agent against CYP and R-induced liver and pancreatic damage, demonstrating its ability to enhance antioxidant capacity, improve pancreatic function, and modulate inflammatory responses. These findings warrant further investigation into the clinical application of STS in the prevention and treatment of liver and pancreatic disorders.

## Data Availability

No datasets were generated or analysed during the current study.

## Abbreviations

MAPK: Mitogen-Activated Protein Kinase
ERK: Extracellular Signal-Regulated Kinase
JNK: c-Jun N-terminal Kinase
CYP: Cyclophosphamide
Nrf2: Nuclear factor erythroid 2-related factor 2
qPCR: Quantitative Polymerase Chain Reaction
MDA: Malondialdehyde
ROS: Reactive Oxygen Species
AST: Aspartate Aminotransferase
ALT: Alanine Aminotransferase
R: Ionizing Gamma Radiation
GSH: Glutathione
STS: Sodium Thiosulfate
GST: Glutathione S-Transferase
H_2_S: Hydrogen Sulfide
